# Stability of retained austenite in high carbon steel under compressive stress: an investigation from macro to nano scale

**DOI:** 10.1038/srep34958

**Published:** 2016-10-11

**Authors:** R. Hossain, F. Pahlevani, M. Z. Quadir, V. Sahajwalla

**Affiliations:** 1 Centre for Sustainable Materials Research and Technology, School of Materials Science and Engineering, UNSW, Australia; 2 Electron Microscopy Unit, Mark Wainwright Analytical Centre, UNSW, Australia; 3 Microscopy and Microanalysis Facility, John de Laeter Centre, Curtin University, Australia

## Abstract

Although high carbon martensitic steels are well known for their industrial utility in high abrasion and extreme operating environments, due to their hardness and strength, the compressive stability of their retained austenite, and the implications for the steels’ performance and potential uses, is not well understood. This article describes the first investigation at both the macro and nano scale of the compressive stability of retained austenite in high carbon martensitic steel. Using a combination of standard compression testing, X-ray diffraction, optical microstructure, electron backscattering diffraction imaging, electron probe micro-analysis, nano-indentation and micro-indentation measurements, we determined the mechanical stability of retained austenite and martensite in high carbon steel under compressive stress and identified the phase transformation mechanism, from the macro to the nano level. We found at the early stage of plastic deformation hexagonal close-packed (HCP) martensite formation dominates, while higher compression loads trigger body-centred tetragonal (BCT) martensite formation. The combination of this phase transformation and strain hardening led to an increase in the hardness of high carbon steel of around 30%. This comprehensive characterisation of stress induced phase transformation could enable the precise control of the microstructures of high carbon martensitic steels, and hence their properties.

For many years, high carbon steels have proved useful for industrial application in extreme operation conditions due to their hardness, strength and relatively low cost compared to high alloy steels. High carbon martensitic steels are favoured when high abrasion resistance is required. These steels contain plate and lath martensite which is formed from austenite during quenching, although this transformation is rarely complete, and some austenite remains. An inverse relationship exists between the strength of the martensite formed in the quenching process and the amount of residual austenite; the martensite’s strength increases as the amount of retained austenite decreases. Hence retained austenite is generally considered deleterious in low-carbon steel^1^,^2^,^3^,^4^,^5^. However, the retained austenite can subsequently be transformed to the more stable martensite phase with the application of high stresses and temperatures; thereby increasing the toughness and ductility of the substrate. This means that under extreme operating conditions, when the pressures on the substrate, and the temperature to which is it exposed, are high enough, the transformation of retained austenite will be triggered, thereby achieving additional work hardening of the steel *in-situ*. This work hardening may be very desirable in industrial applications in which the steel’s surface wears due to the application of stresses, but the material remains hard due to the transformation of retained austenite to martensite. Depending upon its chemical composition, retained austenite can be metastable phase and will transform to martensite by passing the phase transformation barrier energy. Martensitic transformation is achieved by the cooperative shear movement of atoms; applied compressive stress involving compression deformation aids the transformation^6^. If the steel is subjected to high compression or if it is heated to temperatures less than the martensitic transformation temperature, the retained austenite is transformed into martensite. Depending on the chemical composition of steel and temperature, various deformation mechanisms such as mechanical twinning; γ → α´ and γ → ɛ, martensitic transformations can occur under external loading^7^. Here, γ, α´, ɛ are denoted for austenite, BCC/BCT martensite and HCP martensite, respectively. It is very important to measure the amount of retained austenite and its stability to optimise the processing conditions of high carbon martensitic steel for dimensional stability and strength for high abrasion working environments. However, very little attention has been paid to the stability of retained austenite in high carbon steels, despite the reliance of their martensitic microstructure for their strength and hardness and the potential, as discussed above, for the stress induced transformation of retained austenite to improve the steel’s performance.

This study focused on high carbon martensitic steel for use as a wear resistant material in industry. The mechanical stability at the micro and nano level and its stress-induced martensite transformation behaviour has been investigated by Optical microscopy, X-ray diffraction, Electron backscattering diffraction and Nano-indentation technique. In addition, the strain hardening effect on the overall hardness of the high carbon steel was investigated by micro and nano-hardness analyses. Identifying the volume percentage of each phase under compressive stress as well as phase transformation steps, is essential to characterise high carbon steel as a wear resistant material for extreme operating conditions.

## Experimental procedure

Industrial grade high-carbon steel with the chemical composition shown in Table 1 was investigated. It contained a mixture of martensite and a large amount of retained austenite (59–60%). A sample for optical microscopy observation was cut, ground and polished according to standard procedures and then etched in a 2% Nital solution. For the compression tests, 12 samples were cut into small sizes (4 *mm* × 4 *mm* × 4 *mm*) using a diamond cutter at a very slow speed (0.01 mm/sec) to minimise both the heat effects and shear stress, thereby ensuring that retained austenite was not inadvertently transformed as the sample were prepared. Standard metallographic wet grinding and polishing methods were used to prepare the samples for X-ray analysis. A PANalytical Empyrean XRD instrument was used with unfiltered Co-Kα radiation at 45 kV and 40 mA current for quantitative XRD to measure the volume fraction of phases from a 2θ spectrum that was acquired at a step size of 0.0260 over an angular range of 40° to 130°. The compression deformation experiment was performed at room temperature with an Instron 8510 instrument operating at 0.10 mm/min cross-head speed over a loading pressure of 200 MPa to 2500 MPa.

After X-ray diffraction characterization, an orientation microscopy investigation of transformed austenite and martensite was conducted by electron back-scattered diffraction (EBSD) technique, using an Oxford system attached with a Carl Zeiss AURIGA^®^ CrossBeam^®^ field emission gun scanning electron microscopy (FEG SEM) workstation. To determine the steel’s quantitative carbon and manganese content an electron probe micro-analysis (EPMA) was conducted using a Jeol JXA 8500F Hyper probe machine. Nano-indentation tests were carried out in load control mode on a TI 900 Hysitron Tribolab system at varying loads up to 8000 μN with a Berkovich three-sided pyramidal diamond tip indenter (nominal angle of 65.3° and radius of 200 nm). As the varying loads were applied to the austenitic phase, microstructural images helped to identify the resulting phase transformation phenomena at the nanoscale. To obtain the hardness profile of the compression induced material, nano-indentations were made in a 3 × 3 matrix with a uniform load of 8000 μN. Conventional micro Vickers hardness tests were also performed to obtain macroscopic strength at 0.2 HV load.

## Results and Discussion

### Mechanical stability in Micro scale

**Figure 1:**
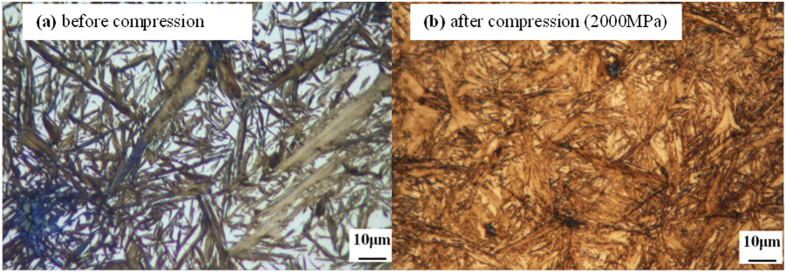
Optical Micrograph of the sample showing effect of compression on retained austenite (Light areas) and martensite (Dark areas) morphology [magnification-100X]. (**a**) Sample without compression deformation. (**b**) Sample with compression deformation (2000 MPa).

**Figure 2:**
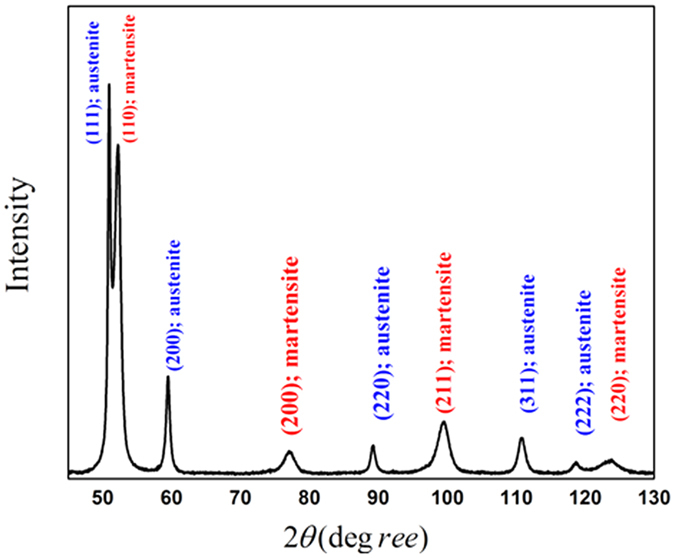
XRD profile of steel without compression deformation.

After heat treatment the structure of the steel was found to contain martensite and retained austenite, which were observed in the optical microscopy images in Fig. 1 and XRD spectrum in Fig. 2. Martensite has two different morphologies: plate and lath martensite. The optical micrographs in Fig. 1 revealed the presence of retained austenite (light areas) with lath and plate-shaped martensite (dark areas). This process is well described in the literature in this field. Two types of retained austenite–blocky and film morphologies–were found in the samples. After heat treatment but prior to compression testing the specimen contained a substantial fraction (59–60%) of retained austenite (Fig. 1a). After compression tests, ranging from 200 MPa to 2500 MPa at room temperature, the amount of retained austenite was found to have reduced significantly. For example, at 2000 MPa it fell to about 18% (Fig. 1b) and at 2500 MPa to below 10%.

All the samples were investigated by XRD. The volume fraction of retained austenite varied within the 59–60% range in all the samples before compression testing. Figure 2 shows a typical XRD profile before deformation, individual diffracting planes correspond to the austenite and martensite phases as labelled.

X-ray analysis was carried out to study the mechanical stability of the retained austenite and other phases on the macro scale under compression deformation. In the following, the volume fraction calculating methods from the XRD spectrum are described. According to the ASTM-E975–13 standard, the volume fraction of each phase can be calculated based on equation (1):


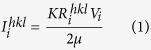


where, 
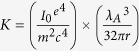



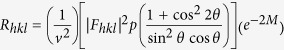



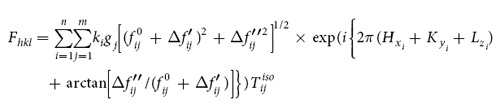



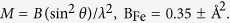


In this equation, 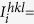 Integrated intensity per angular diffraction peak (hkl) in the i-phase; *I*_0_ = Intensity of the incident beam; *μ *= Linear absorption coefficient for the steel; *e*, *m* =  Charge and mass of the electron; *r* = Radius of the diffractometer; *c* = Velocity of light; *λ* = Wavelength of incident radiation; *v* = Volume of the unit cell; *F*_*hkl*_ = Structure factor which depends on the atomic co-ordinates (*x*_*i*_, *y*_*i*_, *z*_*i*_), the atomic scattering factors 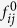 and anomalous dispersion corrections 
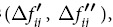
 the isotropic Debye-Waller factors 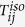 and the influence of occupations *k*_*i*_ and replacements *g*_*j*_; *p* = Multi*p*licity factor of the (*hkl*) reflection; *θ* = Bragg angle; *e*^−2*M*^ = D*e*bye-Waller or temperature factor which is a function of θ; *V*_*i*_ = Volume fraction of i -plane. The constant *K* is composed of various physical properties of the material. The terms in the *R* factor involve the unit cell volume, structure factor, crystallographic multiplicity factor, Lorentz polarization factor and the temperature factor.

Therefore, for a steel containing FCC austenite (*γ*), BCC-martensite (*α*) and HCP-martensite (*ε*), total volume of the phases can be written as:


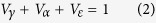


Based on equation 1 individual volume fractions of each phase can be calculated using equation (3),


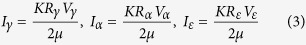


In a specific X-ray diffraction plane 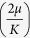 is a constant, therefore,


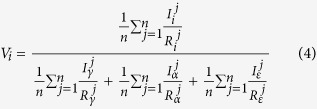


where *i* = *γ*, *α*, *ε* and *n* = number of peaks examined by the X-ray diffraction^8^.

**Figure 3:**
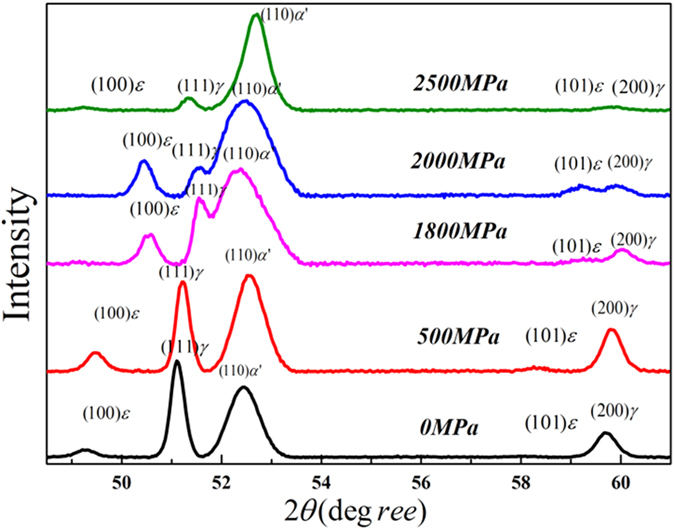
X-ray diffraction scan of high carbon steel compressed at room temperature showing the phase transformation of retained austenite to martensite. Here *γ*, *ε*, *α*′ is denoted for the retained austenite, hcp martensite and bcc/bct martensite respectively.

Figure 3 shows XRD spectrums before and after compression testing. After compression, there are variations in the peak intensities and their positions due to the phase transformations and the sizes of crystal structures. Before compression testing, the sample showed austenitic peaks at (111)_*γ*_ and (200)_*γ*_ diffraction positions. As the compressive stress was increasing the austenite peak started decreasing and the martensite peak at (110)_*α*'_ started increasing. Also, the peak position shifted with the stress. These XRD phenomena correspond closely to the transformation of austenite to martensite as a result of compressive stresses. According to the literature, the peak shift occurs due to lattice expansion during the transformation of retained austenite to martensite^9^. Before compression deformation, the ε–martensite peaks were insignificant as this structure was formed by heat treatment, Fig. 3, 0 MPa spectrum. The literature stated that at least 3% of ε–martensite is necessary to reveal this phase with XRD^10^. After compression deformation the (100)_*ε*_ and (101)_*ε*_ peaks became apparent, indicating that the formation of hexagonal ε–martensite phase occurred during deformation (Fig. 3).

**Figure 4:**
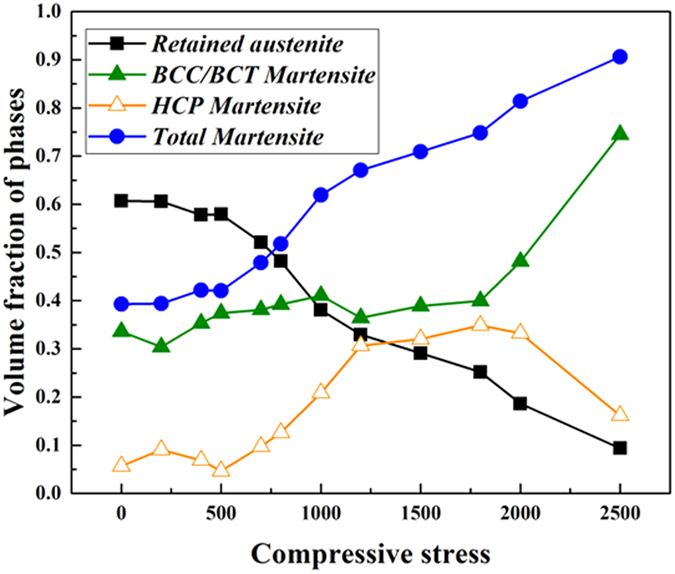
Effect of compressive stress on the volume fraction of phases calculated from XRD pattern. The deformation induced samples show decreasing retained austenite fraction and increasing BCT martensite with increasing stress. The HCP martensite fraction shows increased and decreased pattern. Overall martensite volume fractions under different compression deformations are also shown.

Figure 4 shows the effect of compressive stress on the volume fractions of the phases. From the X-ray spectrum, 60% retained austenite was measured after loading in the 0–200 MPa range. At the highest loads of 2500 MPa, the content of retained austenite decreased to below10%. At low compressive stresses (0–500 MPa) the retained austenite fraction does not vary significantly since the compressive stresses are less than the corresponding activation energy required for phase transformation. The relevant literature show that when the retained austenite attains sufficient energy from the induced compression, randomly spaced overlapping stacking faults create ε-martensite^11^,^12^. The α′-martensite phase nucleates at the intersections of shear bands, i.e. dislocation pile-ups on closely spaced slip planes^12^,^13^,^14^. As a result of the transformation phenomenon, the amount of retained austenite decreases gradually as the stress applied is increased. In these spectrums, the content of *α*′-martensite is considered steady up to a 1500 MPa load, although there is a small increment (0.34% to 0.40%) between 200 MPa to 1500 MPa compressive stresses. When the applied pressure exceeded a 1500 MPa load, the volume fraction of *α*′–martensite increased abruptly to 75% (Fig. 4a). This may have occurred as the structures obtained sufficient energy for transformation from retained austenite to *α*′–martensite over a 1500 MPa load.

The small amount of untransformed retained austenite was stable and unchanged at 3000 MPa compression because it was encapsulated in the newly formed martensite plates which imposed additional compressions on the retained austenite, thereby restricting further transformation. Thus the mechanical stability of the austenite phase was achieved in the steel sample. Above the 2000 MPa compression load the change in ε-martensite content was not so significant but the *α*′-martensite content increased noticeably. This indicates that the transformation process involves both *γ* → *ε* and *γ* → *α*′ transformations and enables strain hardening.

**Figure 5:**
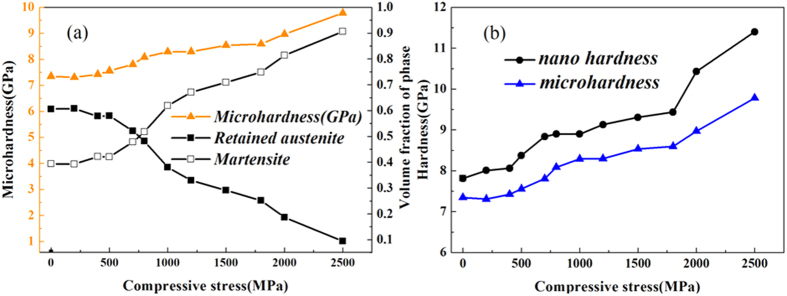
(**a**) Micro hardness profile and phase volume fraction of high carbon steel under compressive stress. (**b**) Micro and nano hardness profile of high carbon steel samples under compressive stress.

It is well known that sample hardness increases with increasing martensitic volume fractions. According to the literature, the martensitic structures act as a barrier to dislocation movements and hence deformations^15^,^16^. Figure 5 shows the results of samples in nano and micro-scale hardness vs. compressive stress tests. When the increasing compressive stress reached 1500 MPa, a sudden increase in hardness occurred, and exceeded 9.78 GPa (Fig. 5a). This correlated closely with the proportion of martensite in the structure and the transformation of retained austenite to martensite, as indicated in Fig. 4. Before compression deformation the hardness was measured at 745HV (7.31 GPa) in micro-hardness testing and 7.81 GPa in nano-hardness testing. This discrepancy becomes apparent in the plot in Fig. 5b, whereby the nano-hardness measurement shows higher hardness values than the micro-hardness measurements. It has been reported that the nano-hardness data show 10–30% higher values than the Vickers hardness data^17^. This is because of the involvement of different deformation mechanisms in these two tests. Briefly, in nano-hardness testing due to concentrated stress on a small area, local strain hardening effects are greater than in micro-hardness testing. Given this explanation, the ratio of Vickers hardness to nano-hardness for the compressed sample is sufficiently consistent to make the hypothesis that the compression deformation of the material resulted in local strain hardening which significantly enhanced the mechanical stability of the high carbon steel. As such, the hardness of the steel was some 30% higher than the hardness of the original steel sample.

**Figure 6:**
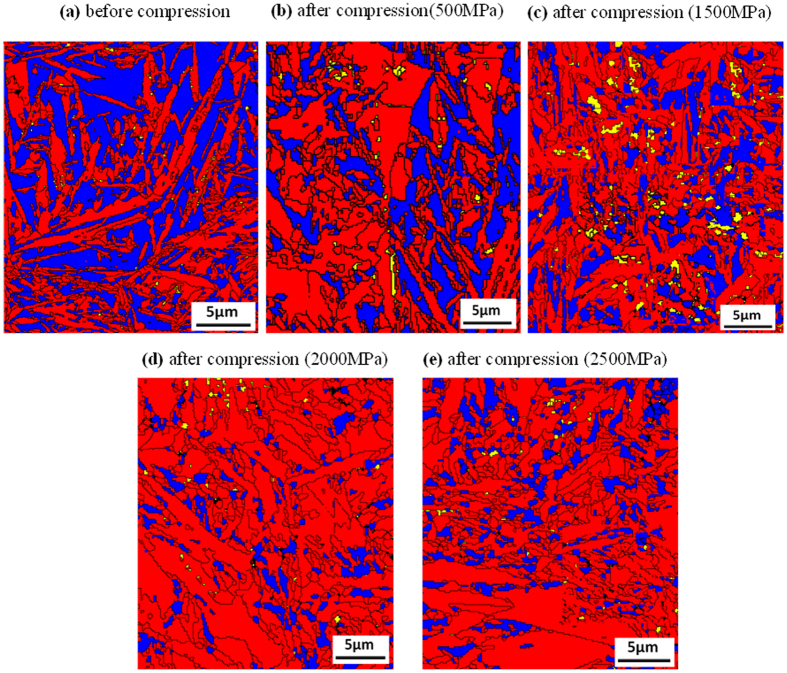
EBSD patterns of studied materials (**a**) without compression and compressed at (**b**) 500 MPa, (**c**) 1500 MPa, (**d**) 2000 MPa and (**e**) 2500 MPa. Different phase has different colours (blue- retained austenite; red- *α*′ martensite and yellow- ε martensite).

Figure 6 shows a series of EBSD measured phase maps that are taken from the undeformed and deformed samples after various loading conditions (500 MPa, 1500 MPa, 2000 MPa and 2500 MPa). In these maps, both *α*′ martensite is plotted as red, ε martensite phases are plotted in yellow, and the retained austenite is plotted in blue. The black lines on the maps represent the boundaries across them where misorientation is over 15°. Overall, the retained austenite grains in Fig. 6a,b are larger than those in Fig. 6c–e. Therefore, the grain size of the retained austenite decreases with increasing compressive load. This is understandable given the well-established theories of crystal plasticity^18^ that state that deformation creates new boundaries and thus divides the original grains into smaller sizes. Those new boundaries are composed of dense distributions of dislocations^19^. An overall comparison between these maps shows a variation in the area fractions of retained austenite. From 500 MPa compression, a reduction in the fraction of retained austenite was observed, when compared to an uncompressed sample (see Fig. 6a,b). Therefore, the transformation begins occurring as early as 500 MPa or earlier. With further load increments, to 1500 MPa–2000 MPa, the reduction of retained austenite becomes more apparent (see Fig. 6c,d) and finally in the 2500 MPa deformed sample, the fraction is lowest. In this sample, the size of the retained austenite is also the smallest. If the grain size of the retained austenite decreases, the stacking fault energy increases^20^,^21^. The martensite phase nucleates at the intersection of shear bands which are created by the overlapping of stacking faults on the austenite planes during deformation. As the SFE increases, it acts as a barrier to the further transformation of retained austenite which makes the finer grain sized retained austenite mechanically more stable than the coarser grained^22^. The distribution of the ε martensite phase indicates that at the early stages of plastic deformation, up to 1500 MPa, HCP martensite formation dominates. This is in line with Fig. 4 which indicates the percentage of each phase during different plastic deformation stages.

**Figure 7:**
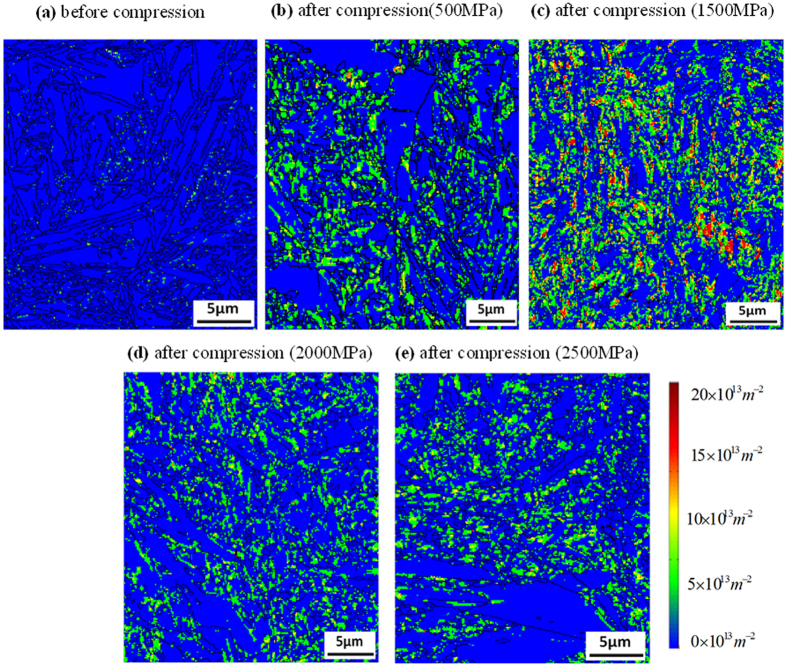
EBSD measured KAM plots showing dislocation density in martensite and austenite structures in samples (**a**) before compression and after compression with (**b**) 500 MPa, (**c**) 1500 MPa, (**d**) 2000 MPa and (**e**) 2500 MPa load. Misorientation boundaries over 15° are plotted in black lines.

It should be noted that these EBSD scan were conducted with a pattern binding of 1 × 1, with an integration number of frames of 10 for 15 kV and that the step size chosen was less than 100 nm. These refined and high resolutions EBSD scans make it possible to detect ε-martensite and to identify the relationship between ε and *α*′ martensite formation and plastic deformation. In the stress inducted martensite formation mechanism, thin martensite plates form first and then other thin plates eventually nucleate near the first ones. Then during the growth process, the remaining austenite is entrapped between individual martensite plates. The EBSD investigation in this study shows the overall area fractions of the two martensite types and so assists in determining their growth in relation to the applied stresses. ε-martensite and *α*′−martensite have distinctively different dislocation contents and this can be displayed by Kernel average misorientation (KAM) plots^23^ (see Fig. 7), which provide additional information on lattice distortions and deformation localizations^24^. From the EBSD data KAM can be obtained from the average misorientation around a measurement point in relation to a defined set of the nearest neighbouring points. Therefore, a high KAM value indicates high dislocation density^25^,^26^,^27^. In this analysis neighbouring pixels with a misorientation angle lower than a threshold of 5° are taken to exclude other boundaries (eg. grain boundary, special feature boundary, etc). The KAM maps in Fig. 7 correspond the phase maps in Fig. 6. These plots display the change in dislocation content in the martensite and austenite structure between pre- and post-compression samples. The grain boundaries are plotted black. The highly distorted regions following compression are near the grain boundaries of the *α*′ martensite phase, the locations for the nucleation of ε-martensite. This study also reveals that more ε martensite than *α*′ martensite forms at the beginning of the plastic deformation (up to 1500 MPa). Later, at compression stresses reach and exceed 2000 MPa *α*′ martensite formation was observed. These observations are made based on dislocation contents (blue zone with lower dislocation density), which are also supported by the XRD data in Figs 3, 4 and 6. At the early stages of compression *γ* → *ε* transformation dominates while at the higher compression stresses *γ* → *α*′ transformation prevails.

**Figure 8:**
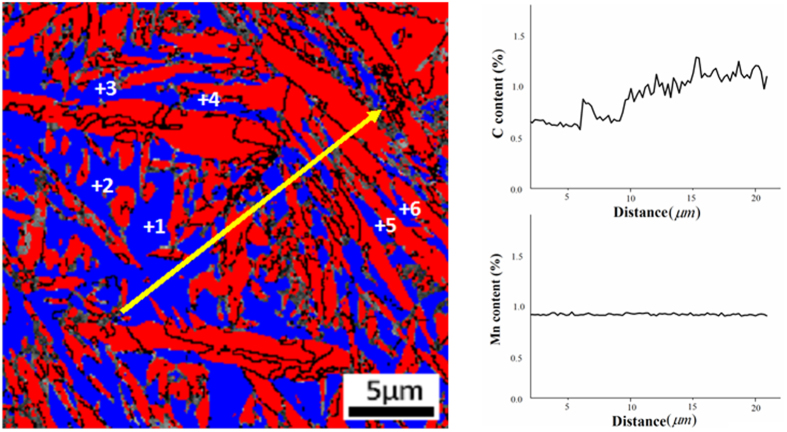
(**a**) EBSD phase map showing the positions of EPMA line scan and spot in the austenite and martensite regions and (**b**) C and Mn profile along the measurement line.

Another factor that influences the retained austenite to martensite transformation is the stacking fault energy (SFE). The stacking fault energy is related to the C and Mn content and therefore a carbon content measurement in the retained austenite was conducted using EPMA. Figure 8(b) shows EPMA line measurement plots of C and Mn on the retained austenite and martensite regions in the sample. The austenite grains that do not contain martensite phase have a homogeneous C distribution with an average C content of 0.65 wt.%. In contrast, the austenite grains that are surrounded by martensite are carbon rich (0.8–1.0 wt%). This indicates of C partitioning from the martensite phase to the austenite phase^26^. The Mn content of the austenite grains does not change very much, based on the spatial distribution of martensite phase, and therefore a uniform Mn profile is measured in Fig. 8. It is known that SFE increases with carbon content and it can be estimated from the equation described in ref. 27, 28, 29, 30, 31, 32. The average C and Mn content in austenite phases (based on EPMA data) and the calculated SFEs are listed in Table 2; it was found that the SFE varies within the 13–18 mJm^−2^ range. The literature shows the formation of martensite occurs below18 mJm^−2^ SFE^28^,^33^,^34^. Therefore, in the studied samples, ε martensite formation may be facilitated by the formations of stacking faults during the early deformation stages. It is pertinent to note that literature also shows that deformation induced stacking faults lead to the formation of shear bands, and where shear bands from several glide systems intersect, the nucleation of α′ martensite is initiated^22^,^34^.

### Mechanical stability in Nano-scale

**Figure 9:**
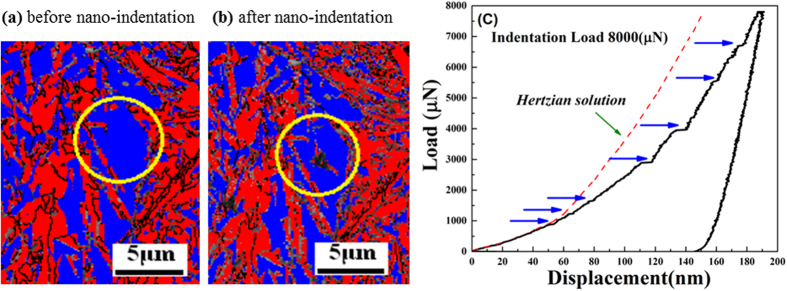
The EBSD phase map of austenite grains in blue and martensite grains in red (**a**) before nano-indentation (**b**) after nano-indentation. (**c**) Nano-indentation load displacement curve on individual retained austenite grain. The blue arrows are indicating discontinuous displacement bursts during nano-indentation. The red dashed line represents the calculated Hertzian elastic contact solution.

To observe the stress induced phase transformations EBSD scanning was carried out, before and after the nano-indentation experiments, as per the data shown in figure Fig. 9. Figure 9a shows the distribution of the austenite and martensite phases prior to nano-indentation. Then the austenite area was located and the nano-indentation experiment was carried out. Another EBSD scan was conducted on the same area (in Fig. 9b). Thus, it was demonstrated that the formation of the martensite phase occured as a result of the nano-indentation. In the load displacement curve in Fig. 9c it was found that the indentation penetration depth reached up to 181 nm when the load increased 8000 μN. At the initial loading stages displacement was continuous until ~55 nm distance, then discontinuous displacement bursts appeared. These points are marked by blue arrows on the load-displacement curve. Here the bursts are obvious since the acceleration of martensitic transformation is quite swift. During these events the indenter tip reacts at an exceedingly faster rate than the pre-set experimental rate to maintain a constant loading rate. Therefore, after every burst, the curve deviates from the original trajectory, which is shown with a red dashed line in Fig. 9c. This serves as an indication of the strain hardening effect due to phase transformation and associated dislocation movements.

Broadly, the appearance of discontinuous displacement bursts in crystalline metals can be associated with certain factors, for instance; dislocation nucleation and propagation; crack formation or phase transformation^35^,^36^,^37^. Studies have shown that if there are simultaneous bursts occurring, the first burst corresponds to dislocation nucleation^38^ and then dislocation pile-up, dislocation movement and phase transformation will follow.

In Fig. 9c, the first burst occurs with a small penetration depth of around ~57 nm. It was considered that the high-stress zone beneath the indenter is free of mobile dislocations, instead of grain boundaries. Accordingly, the first burst occurred at the beginning of the deformation and is interpreted as the result of dislocation nucleation in the retained austenite grain. This correlates with the dislocation mapping for the bulk sample in which dislocation density starts increasing at the beginning of the plastic deformation (see Fig. 7). In order to fully understand the phase nature of retained austenite, the maximum shear stress *τ*_max_ at the elastic zone can be compared with the theoretical value when the first burst occurred. This can be calculated by equation (5)^39^:


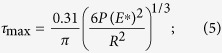


where, 
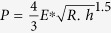


*R* = Radius of the indentation tip; *h* = Corresponding indentation depth

E^*^ = Effective modulus of the indentation can be written as:
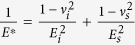
, where, *E* is the Young’s modulus, *ν* is the Poisson’s ratio and the subscript i and s represent the indenter and specimen respectively.

Based on equation (5), *τ*_max_ was calculated to be 21.45 GPa (Fig. 7c) which is about 1/3 to 1/4 of the theoretical shear modulus of austenite phase (75–81 GPa). The induced stress is within the theoretical strength of polycrystalline metals (G/30 to G/5 of theoretical shear modulus). Therefore, it is plausible to assume that the first burst is associated with dislocation nucleation^38^,^40^,^41^,^42^,^43^. The second burst that appeared in the high carbon steel sample, was generated by dislocation movement. Consequent bursts represent the outbreak of strain-induced phase transformation from the retained austenite to martensite reaction^40^ in the nano-scale. The bursts in Fig. 7 starts almost at the same pressure for both the indentations (Fig. 7a,b) which shows that the metastable retained austenite grain starts transforming into martensite when the specific activation energy is crossed as an increasing load is applied load during indentation. By contrast, although multiple indentations were carefully applied to the martensite grain in similar nano-indentation experiments, no noticeable bursts in the load curve were observed. This indicates that phase transformations did not occur in the martensite during the indentation process.

## Conclusion

This work presents the first investigation at both the macro and nano level of the mechanical stability of retained austenite in high carbon steel samples under compressive stress, using a combination of OM, EBSD, XRD, EPMA, micro-indentation and nano-indentation tests. OM and EBSD patterns showed a significant reduction in the grain size and volume of retained austenite at increased compressive stresses. The retained austenite to martensite transformation was characterized in terms of the volume fractions of the phases formed in the deformation and transformation mechanism through the analysis of X-ray diffraction patterns. Quantitative analyses by XRD and dislocation density plots revealed that larger amounts of ε–martensite formed compared to that of α′-martensite at lower loads. The volume percentage of α′-martensite increased as the compression load increased. The transformation process involved i.e., *γ* → *ε* and *γ* → *α*′ efficiently increases the mechanical stability of the steel. Consistent results of micro-hardness and nano-hardness tests showed a positive correlation between compressive stress and the increasing hardness of the material, which can be attributed to strain hardening and phase transformation.

In our industrial grade high carbon steel samples, multiple discontinuous displacement bursts were observed in the load displacement curve by imposing nano-indentation stresses on retained austenite grains. These results demonstrate that the first burst was associated with dislocation nucleation and that subsequent bursts were the result of the progressive transformation of austenite to martensite. We have established, and have comprehensively described, how external compressive stress; increased dislocations density; smaller grain size and additional compressive stress caused by the martensitic transformations can improve the hardness and mechanical stability of high carbon steel. Such understanding is critical for controlling the microstructures of high carbon steels and, so, for opening up new industrial applications for these relatively cost-effective steels.

## Additional Information

**How to cite this article**: Hossain, R. *et al*. Stability of retained austenite in high carbon steel under compressive stress: an investigation from macro to nano scale. *Sci. Rep*. **6**, 34958; doi: 10.1038/srep34958 (2016).

## Figures and Tables

**Table 1:** Chemical composition of the investigated high carbon steel.

Elements	C	Si	Mn	Cr	Ni	Cu	Mo
wt%	0.99	0.23	0.98	0.65	0.06	0.18	0.02

**Table 2:** (a) EPMA measured C and Mn contents of the labelled spots in the phase map in Fig. 8a,b calculated corresponding stacking fault energies.

Location marks	C	Mn	SFE(mJm^−2^)
1	0.56	0.95	12.39
2	0.61	0.93	13.01
3	0.65	0.96	14.67
4	0.67	0.91	15.37
5	0.91	0.93	18.09
6	0.89	0.97	16.89

## References

[b1] Scott D. & Blackwell J. The effect of some manufacturing variables on the performance of high-speed tool-steel ball bearings. Wear 18, 19–28 (1971).

[b2] Alley E. S. & Neu R. W. Microstructure-sensitive modeling of rolling contact fatigue. International Journal of Fatigue 32, 841–850 (2010).

[b3] Hengerer F., Nierlich W., Volkmuth J. & Nützel H. Dimensional stability of high carbon bearing steels. Ball Bearing Journal 231, 26–31 (1988).

[b4] Zaccone M. Fatigue and Strain-Hardening of High Carbon Martensite–Austenite Composite Microstructures. Heat Treatment'87, 93–101 (1987).

[b5] Zaccone M. & Krauss G. Elastic limit and microplastic response of hardened steels. Metallurgical Transactions A 24, 2263–2277 (1993).

[b6] Tamura I. Deformation-induced martensitic transformation and transformation-induced plasticity in steels. Metal Science 16, 245–253 (1982).

[b7] Sherby O. D., Wadsworth J., Lesuer D. R. & Syn C. K. Revisiting the structure of martensite in iron-carbon steels. Materials transactions 49, 2016–2027 (2008).

[b8] De A. K., Murdock D. C., Mataya M. C., Speer J. G. & Matlock D. K. Quantitative measurement of deformation-induced martensite in 304 stainless steel by X-ray diffraction. Scripta Materialia 50, 1445–1449 (2004).

[b9] Mao H. K., Bassett W. A. & Takahashi T. Effect of pressure on crystal structure and lattice parameters of iron up to 300 kbar. Journal of Applied Physics 38, 272–276 (1967).

[b10] Stanford N., Dunne D. P. & Monaghan B. J. Austenite stability in Fe–Mn–Si-based shape memory alloys. Journal of alloys and compounds 430, 107–115 (2007).

[b11] Angel T. Formation of martensite in austenitic stainless steels-effects of deformation, temperature, and composition. Journal of the iron and steel institute 177, 165-& (1954).

[b12] Brooks J., Loretto M. & Smallman R. Direct observations of martensite nuclei in stainless steel. Acta Metallurgica 27, 1839–1847 (1979).

[b13] Suzuki T., Kojima H., Suzuki K., Hashimoto T. & Ichihara M. An experimental study of the martensite nucleation and growth in 18/8 stainless steel. Acta Metallurgica 25, 1151–1162 (1977).

[b14] Brooks J., Loretto M. & Smallman R. *In situ* observations of the formation of martensite in stainless steel. Acta Metallurgica 27, 1829–1838 (1979).

[b15] Litwinchuk A., Kayser F., Baker H. & Henkin A. The Rockwell C hardness of quenched high-purity iron-carbon alloys containing 0.09 to 1.91% carbon. Journal of Materials Science 11, 1200–1206 (1976).

[b16] Krauss G. Martensitic transformation, structure and properties in hardenable steels. Metallurgical Society AIME 229–248 (1978).

[b17] Qian L., Li M., Zhou Z., Yang H. & Shi X. Comparison of nano-indentation hardness to microhardness. Surface and Coatings Technology 195, 264–271 (2005).

[b18] Valiev R. Z. & Langdon T. G. Principles of equal-channel angular pressing as a processing tool for grain refinement. Progress in Materials Science 51, 881–981 (2006).

[b19] Afrin N., Quadir M., Xu W. & Ferry M. Spatial orientations and structural irregularities associated with the formation of microbands in a cold deformed Goss oriented Ni single crystal. Acta Materialia 60, 6288–6300 (2012).

[b20] Jun J.-H. & Choi C.-S. Variation of stacking fault energy with austenite grain size and its effect on the M S temperature of γ → ε martensitic transformation in Fe–Mn alloy. Materials Science and Engineering: A 257, 353–356 (1998).

[b21] Misra R., Kumar B. R., Somani M. & Karjalainen P. Deformation processes during tensile straining of ultrafine/nanograined structures formed by reversion in metastable austenitic steels. Scripta Materialia 59, 79–82 (2008).

[b22] Talonen J. & Hänninen H. Formation of shear bands and strain-induced martensite during plastic deformation of metastable austenitic stainless steels. Acta materialia 55, 6108–6118 (2007).

[b23] Calcagnotto M., Ponge D., Demir E. & Raabe D. Orientation gradients and geometrically necessary dislocations in ultrafine grained dual-phase steels studied by 2D and 3D EBSD. Materials Science and Engineering: A 527, 2738–2746 (2010).

[b24] Saraf L. Kernel average misorientation confidence index correlation from FIB sliced Ni-Fe-Cr alloy surface. Microscopy and Microanalysis 17, 424–425 (2011).

[b25] Li H., Hsu E., Szpunar J., Utsunomiya H. & Sakai T. Deformation mechanism and texture and microstructure evolution during high-speed rolling of AZ31B Mg sheets. Journal of materials science 43, 7148–7156 (2008).

[b26] Gouné M., Danoix F., Allain S. & Bouaziz O. Unambiguous carbon partitioning from martensite to austenite in Fe–C–Ni alloys during quenching and partitioning. Scripta Materialia 68, 1004–1007 (2013).

[b27] Dinsdale A. T. SGTE data for pure elements. Calphad 15, 317–425 (1991).

[b28] Allain S., Chateau J.-P., Bouaziz O., Migot S. & Guelton N. Correlations between the calculated stacking fault energy and the plasticity mechanisms in Fe–Mn–C alloys. Materials Science and Engineering: A 387, 158–162 (2004).

[b29] Yang W. & Wan C. The influence of aluminium content to the stacking fault energy in Fe-Mn-Al-C alloy system. Journal of materials science 25, 1821–1823 (1990).

[b30] Ishida K. & Nishizawa T. Effect of alloying elements on stability of epsilon iron. Transactions of the Japan Institute of Metals 15, 225–231 (1974).

[b31] Huang W. An assessment of the Fe-Mn system. Calphad 13, 243–252 (1989).

[b32] Adler P., Olson G. & Owen W. Strain hardening of Hadfield manganese steel. Metallurgical and Materials Transactions A 17, 1725–1737 (1986).

[b33] Dick A., Hickel T. & Neugebauer J. The Effect of Disorder on the Concentration‐Dependence of Stacking Fault Energies in Fe1‐xMnx–a First Principles Study. steel research international 80, 603–608 (2009).

[b34] He B., Luo H. & Huang M. Experimental investigation on a novel medium Mn steel combining transformation-induced plasticity and twinning-induced plasticity effects. International Journal of Plasticity 78, 173–186 (2016).

[b35] Kim B., Trang T. & Kim N. J. Deformation behavior of ferrite-austenite duplex high nitrogen steel. Metals and Materials International 20, 35–39 (2014).

[b36] Misra R. . Nanomechanical insights into the deformation behavior of austenitic alloys with different stacking fault energies and austenitic stability. Materials Science and Engineering: A 528, 6958–6963 (2011).

[b37] Misra R., Zhang Z., Jia Z., Somani M. & Karjalainen L. Probing deformation processes in near-defect free volume in high strength–high ductility nanograined/ultrafine-grained (NG/UFG) metastable austenitic stainless steels. Scripta Materialia 63, 1057–1060 (2010).

[b38] He B. . Nanoindentation investigation on the mechanical stability of individual austenite grains in a medium-Mn transformation-induced plasticity steel. Scripta Materialia 69, 215–218 (2013).

[b39] Johnson K. Contact Mechanics, Cambridge University Press, Cambridge, 1985.

[b40] Ahn T.-H. . Investigation of strain-induced martensitic transformation in metastable austenite using nanoindentation. Scripta Materialia 63, 540–543 (2010).

[b41] Gouldstone A., Koh H.-J., Zeng K.-Y., Giannakopoulos A. & Suresh S. Discrete and continuous deformation during nanoindentation of thin films. Acta Materialia 48, 2277–2295 (2000).

[b42] Bahr D., Kramer D. & Gerberich W. Non-linear deformation mechanisms during nanoindentation. Acta materialia 46, 3605–3617 (1998).

[b43] Sekido K. . Nanoindentation/atomic force microscopy analyses of ε-martensitic transformation and shape memory effect in Fe–28Mn–6Si–5Cr alloy. Scripta Materialia 65, 942–945 (2011).

